# Copper Nanoparticles Mediated by Chitosan: Synthesis and Characterization via Chemical Methods

**DOI:** 10.3390/molecules171214928

**Published:** 2012-12-14

**Authors:** Muhammad Sani Usman, Nor Azowa Ibrahim, Kamyar Shameli, Norhazlin Zainuddin, Wan Md Zin Wan Yunus

**Affiliations:** 1 Department of Chemistry, Faculty of Science, Universiti Putra Malaysia, UPM Serdang, Selangor 43400, Malaysia; E-Mails: kamyarshameli@gmail.com (K.S.); hazlin@science.upm.edu.my (N.Z.); 2 Materials and Energy Research Centre, Karaj 317-798-3634, Iran; 3 Department of Chemistry, Faculty of Defence Science and Technology, National Defence University of Malaysia, 57000 Kuala Lumpur, Malaysia; E-Mail: wanmdzin@upnm.edu.my

**Keywords:** chitosan, copper sulphate, copper nanoparticle, chemical reduction method

## Abstract

Herein we report a synthesis of copper nanoparticles (Cu-NPs) in chitosan (Cts) media via a chemical reaction method. The nanoparticles were synthesized in an aqueous solution in the presence of Cts as stabilizer and CuSO_4_·5H_2_O precursor. The synthesis proceeded with addition of NaOH as pH moderator, ascorbic acid as antioxidant and hydrazine as the reducing agent. The characterization of the prepared NPs was done using ultraviolet-visible spectroscopy, which showed a 593 nm copper band. The Field Emission Scanning Electron Microscope (FESEM) images were also observed, and found to be in agreement with the UV-Vis result, confirming the formation of metallic Cu-NPs. The mean size of the Cu-NPs was estimated to be in the range of 35–75 nm using X-ray diffraction. XRD was also used in analysis of the crystal structure of the NPs. The interaction between the chitosan and the synthesized NPs was studied using Fourier transform infrared (FT-IR) spectroscopy, which showed the capping of the NPs by Cts.

## 1. Introduction

Recent advances in nanotechnology have made the nanoscience field a hot area of research, making it one of the most researched areas of science in the past two decades. In general, NPs are described as particles having diameter sizes less than or equal to 0.1 µm (100 nm) and with specific properties that depend mainly on their size [1]. NP research has become an area of attraction due their unique superior properties when compared to their bulk materials [2], which results in their wide range of applications in different fields. Some of these properties include; catalytic properties [3,4,5], thermal properties, electrical conductivity, optical properties [6] and biological applications [7]. Their favorable properties are influenced by their high surface energy with a high surface area to volume ratio and relatively small sizes [8]. Syntheses of NPs in polymer media have been promising due to their ease of processing, solubility, less toxicity and also because of the possibility of controlling the growth of the resulting nanoparticles. 

Copper nanoparticle (Cu-NP) synthesis specifically has attracted more interest compared to other NPs’ synthesis because of their useful properties achievable at much less cost than silver and gold [9]. Copper, like other noble metals, exhibits thermal and electrical conductivity, which makes it a candidate in electronic systems [10] and conductive inks [11]. Similarly, it has antimicrobial properties [12] and is readily available. These properties thus make Cu-NP synthesis an attractive area. There are different methods of synthesis of Cu-NPs, including thermal reduction [13], metal-vapor synthesis [14], chemical reduction [15], vacuum vapor deposition [16], radiation methods [17], microemulsion techniques [18], polyol processes [19] and laser ablation [20]. The major limitation in the synthesis is their ease of oxidation [21] to CuO or Cu_2_O during and after preparation, this makes it difficult to synthesis Cu-NPs without providing an inert environment (Ar, N_2_) [22]. Observations indicate a great number of the Cu-NP syntheses are done in an inert atmosphere or under a protective environment [15,16]. 

Chitosan (Cts) polymers are naturally occurring polymers which are obtained by deacetylazation of chitin; and are derived from complex amino polysaccharides by semi-synthetic means. They are cationic polysaccharides composed of β-(1-4)-linked D-glucosamine and *N*-acetyl-D-glucosamine units. They are nontoxic, biodegradable, biocompatible and bioactive polymers with momentous amounts of amine and hydroxyl groups [23]. They are known for their wide range of applications, often being proposed for pharmaceutical, biomedical, and industrial applications. 

The choice of the Cts as stabilizer of the Cu-NPs is because of its ability to chelate metals, which makes a perfect candidate for metal NP synthesis [24]. Generally, the use of bio-polymers as stabilizers for the synthesis of Cu-NPs is gaining momentum because of their availability, biocompatibility and low toxicity. Here in this research, we report a simple synthesis of Cu-NPs via a chemical method under an ambient atmosphere with Cts as stabilizer, hydrazine as the reducing agent and ascorbic acid as an antioxidant [25].

## 2. Results and Discussion

**Figure 1:**
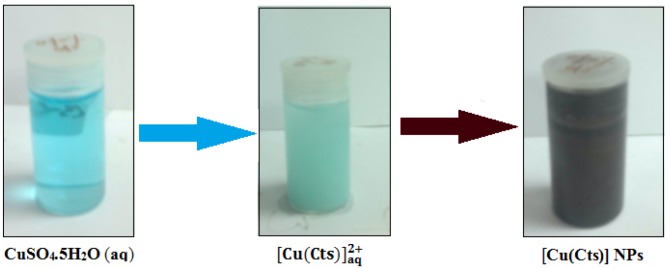
Photographs of the sample at the different stages of synthesis showing colour variations.

The formation of Cu-NPs follows a series of color changes which are not unrelated to the chemical reactions taking place. The dropwise addition of bulk Cu solution to Cts solution resulted in a light blue coloration, which indicates formation of a Cts copper complex [Equation (1), Figure 1]. The ascorbic acid was added at this stage to protect the to be formed NPs from oxidation, it being a good antioxidant [26]. No color change was observed after this addition. A resultant colour change to light green was observed after addition of NaOH. A different observation was however reported by [27,28], whereby according to their reports, a yellow coloration was noticed after the addition of the NaOH which indicated formation of Cu-NPs and Cu_2_O NPs. However, a different trend was observed in this effort as the UV-Vis analysis of sample taken at this stage of the analysis did not show SPR bandwidth, which shows that the NaOH added couldn’t reduce the [Cu(Cts)]^2+^ complex:

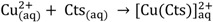
(1)


Thus, the observation stresses the role of the base as pH moderator in this work. The Cu-NPs were though formed following the addition of hydrazine with a quick light brown coloration which turned brick red [Equation (2), Figure 1)]. The formation of NPs was confirmed by UV-Vis absorption analysis. The major role of the Cts in the synthesis is to prevent the aggregation of the nuclei of the NPs by inducing the repulsion forces to overcome the van der Waals forces between the crystals of the individual atoms:

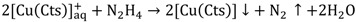
(2)


**Scheme 1:**
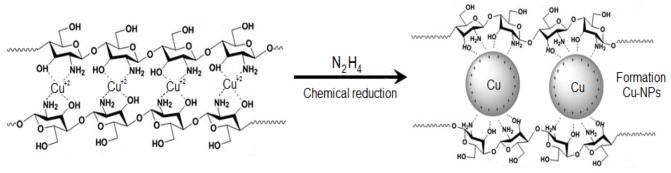
Schematic representation of Cu-NPs embedded in Cts media.

In Scheme 1, chitosan first complexes copper ion, the complex is then reduced to NPs after addition of hydrazine. 

With a core-shell structure, the copper core is surrounded by the hydroxyl and amine groups of the chitosan, which serve as capping, provide stability, prevent Cu_2_O formation and possibly lead to a decrease in the size of the particles. 

### 2.1. UV-Visible Spectroscopy Analysis

**Figure 2:**
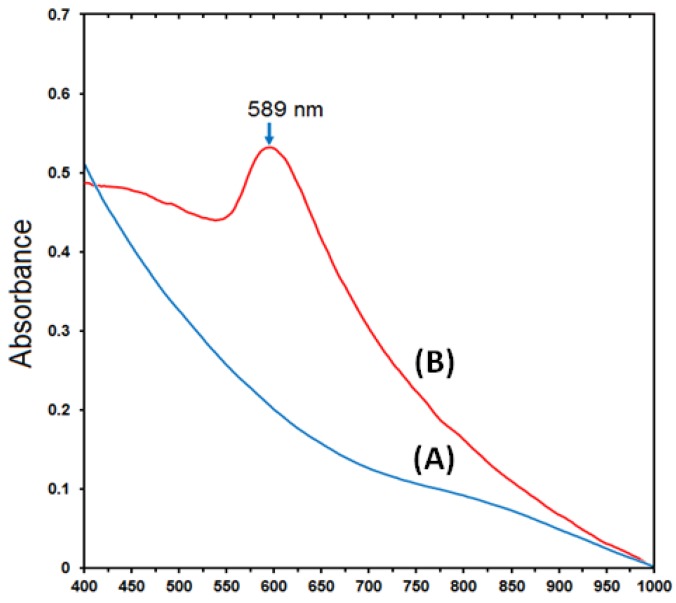
UV-Vis absorption spectra of Cu-NPs in Cts with NaOH (**A**) and 0.1% Cts (**B**).

The synthesis of the Cu-NPs was immediately followed by UV-Vis analysis. The NPs as observed in Figure 2B showed a single SPR peak at 589 nm, which indicates formation of Cu-NPs [7]. The band was observed only after addition of hydrazine, as samples taken prior to the addition did not show any visible peak, which suggests that NPs were not formed after addition of NaOH. Generally, Cu-NPs are known to show an SPR band in the 573–600 nm range [29], as the precise position the SPR band is not known, but rather is determined by certain parameters, such as the solvent used in the synthesis, capping agent, the size and shape of the particles [22]. Additionally, an increase in the bandwidth of the SPR results in a decrease in size of the nanoparticles [30]. However, the SPR of NPs is mostly controlled by certain parameters, such as shape, morphology, size, composition and dielectric environment of the prepared NPs [6]. 

### 2.2. X-ray Diffractmeter

**Figure 3:**
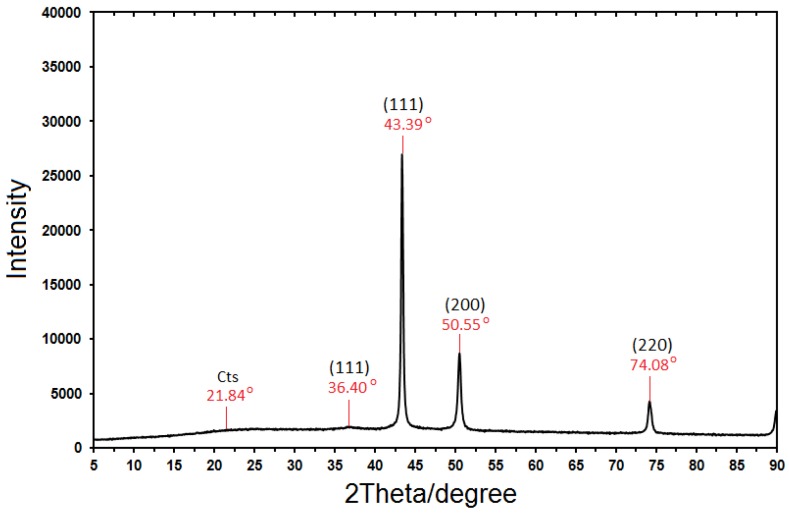
XRD Diffractogram of Cu-NPs synthesized in chitosan media.

From Figure 3, the sample demonstrated a high crystallinity level with diffraction angles of 36.40°, 43.39°, 50.55° and 74.08°, which correspond to the characteristic face centered cubic (fcc) of copper lines indexed at (111), (111), (200) and (220), respectively [22]. The diffraction angle observed at 21.84 is related to the Cts medium [31]. The absence of any noticeable peaks in the pattern resulting from the sample suggests that the sample is free from impurities such as CuO and Cu_2_O. The size of the NPs obtained were estimated to be in the range of 35-75 nm using Debye-Scherrer Equation, which may indicate a high surface area, and surface area to volume ratio of the nano-crystals. The equation is written below:

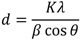
(3)
where K, known as Scherer’s constant (shape factor), ranges from 0.9 to 1.0, *λ* is 1.5418 Å, which is the wavelength of the X-Ray radiation source, β1/2 is the width of the XRD peak at half height and *θ* is the Bragg angle. 

### 2.3. FT-IR Analysis

**Figure 4:**
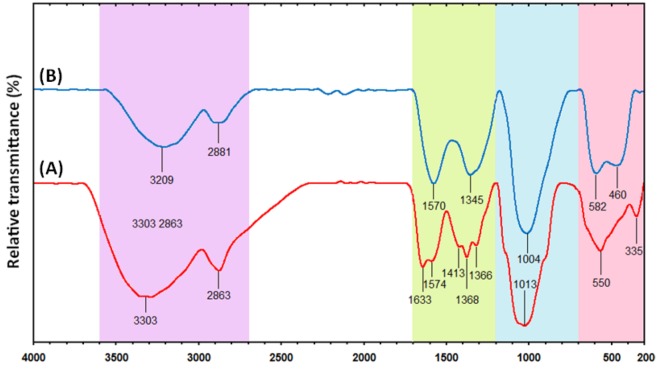
(**A**) FT-IR spectra of pure chitosan and (**B**) Cu-NPs synthesized in chitosan media.

This analysis was conducted to determine the molecular interaction(s) between the Cts and the synthesized NPs. The Cts spectrum in Figure 4A illustrates an amide I C=O vibration band at 1633 cm^−1^ [32]. 

The spectrum also shows transmissions at 3303 cm^−1^ assigned to the overlap of O−H and N−H stretching vibrations [18], 2863 to C−H stretching, 1633 and 1574 cm^−1^ to –NH_2_ bending, 1413, 1368 and 1366 cm^−1^ to C−H bending and 1013 cm^−1^ to −C−O skeletal stretching. A similar trend was observed in the Cts mediated Cu-NPs spectrum (Figure 4B). For instance, a general decrease in band with a blue shift was noticed (from 3303 to 3209, 1574 to 1570, 1368 to 1345 and 1013 to 1004 cm^−1^) [32]. However, a new absorption peak was observed at 460 cm^−1^. The corresponding vibration band is related to the interaction between the Cu-NPs and the Cts media, which indicates a reaction between the Cu-NP surface, and the Cts amino and hydroxyl groups. This suggests that NPs were capped by the polymer [33].

### 2.4. FESEM (Morphology) Analysis

**Figure 5:**
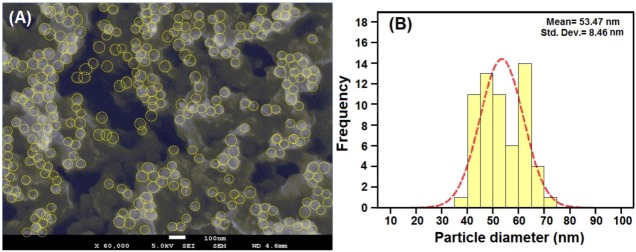
Image of Cu-NPs synthesized in 0.1% Cts media. (**A**) Histogram of embedded chitosan aggregated Cu-NPs distribution (**B**) at 100 nm.

The morphological analysis of the synthesized NPs was done with Field Emission Scanning Microscopy. The samples were prepared by coating with gold to avoid charging effect of the images to be obtained. Figure 5A shows a captured image of the Cu-NPs embedded within the matrix of chitosan, the yellow circular lines indicate the NPs from which the approximately spherical shape of the poly-dispersed copper NPs can be seen. The particles were found to be in the range of 35–75 nm in size as estimated using the Debye-Scherrer equation with data obtained from XRD. This could be due to the temperature at which the NPs were synthesized and the stabilizing effect of the Cts used. Similar observations were reported by [22] where they synthesized Cu-NPs at different temperatures. 

The NPs were observed to be poly-dispersed with a relatively low mean size when synthesized at temperatures of less than 190 °C. More so, the distribution of the NPs is represented by little aggregation of the NPs. This is due to the high surface energy of the individual NPs that is synonymous with their small size [7]. The histogram in Figure 5B showed the average mean sizes and standard deviation of the NPs as 53 ± 8.46 nm, which represents the aggregated copper nano-crystals’ size. A similar observation was noticed by [26], where Cu-NPs were synthesized in polyethylene glycol matrix, which showed little aggregation.

## 3. Experimental

### 3.1. Materials

All chemicals were used as purchased without further purification. CuSO_4_·5H_2_O (99.98%) and ascorbic acid (99.0%) were supplied by Sigma Aldrich (St. Louis, MO, USA), chitosan (600,000–800,000 *M.Wt*) and hydrazine (N_2_H_4_, 98.0%) were supplied by Acros Organics (Geel West, Belgium), NaOH (99.0%) was supplied by R&M Chemicals Ltd (Edmonton, AB, Canada). Double distilled water was used throughout the experiments.

### 3.2. Synthesis of Cu-NPs Mediated in Cts by a Chemical Method

The synthesis of Cu-NPs was done by modification of the method used by Dang *et al.* [26]. Firstly, a blue colored aqueous solution of bulk CuSO_4_·5H_2_O was made by dissolving this substance (0.4 g) in double distilled water (10 mL), then the solution was then added dropwise to a solution (0.1 M acetic acid) of Cts (40 mL) while the color changed from blue to light blue. After stirring and refluxing at 120 °C for about 20 min, 0.05 M ascorbic acid (0.5 mL) was added to the solution. With further stirring for 20 min, 0.6 M NaOH (2 mL) was added to the solution, and no immediate colour change was observed after the addition. However, after stirring for about 30 min, a light green coloration was observed. N_2_H_4_ (0.5 mL) was finally added to give a quick brown coloration which changed to dark red after about 5 min of stirring. The solution was stirred for further 30 min for the reaction to complete and the reaction mixture was allowed to cool at room temperature. The solution was centrifuged at 14,000 rpm for 15 min to obtain the Cu-NPs and the supernatant was discarded. The particles were repeatedly washed to ensure purity. 

### 3.3. Characterizations Methods and Instruments

SPR of the sample was determined using a UV 1650 PC-Shimadzu B UV-visible spectrophotometer (Shimadzu, Osaka, Japan). The spectrum was recorded over a range of 300–700 nm. The crystallographic analysis was done by utilizing XRD. The diffractions of the sample were taken using an XRD-6000 (Shimadzu) X-ray diffraction instrument and the patterns were recorded at a scan speed of 4°/min. The molecular interaction study was done using FT-IR. Spectra of chitosan and the Cu-NPs were recorded with a FT-IR Series 100, 1650 Perkin Elmer spectrophotometer (Perkin-Elmer, Los Angeles, CA, USA), recorded over the range of 200–4000 cm^−1^. The morphological analysis of the as-synthesized NPs was conducted using a Jeol JSM-7600F Field Emission Scanning Microscope JEOL, (Eching b. München, Germany). Prior to the analysis, the samples were coated with a Baltec SCD005 Sputter coater (Bal-tec., Canonsburg, PA, USA).

## 4. Conclusions

Spherical shaped Cu-NPs with average mean sizes in the range of 35–75 nm and an fcc crystal structure were synthesized in a chitosan medium through a simple chemical method. The NPs were characterized by UV-Vis, XRD, FEEM and FT-IR. From the results, it is safe to state that the chemical method of synthesis is the easiest and cheapest method for Cu-NP synthesis with high purity of the NPs obtained. Moreover, the results obtained in this research show the formation of high purity Cu-NPs with chitosan being a major player in stabilization and N_2_H_4_ as the reductant.
